# Identifying self-presentation components among nursing students with unsafe clinical practice: a qualitative study

**DOI:** 10.1186/s12909-023-04486-9

**Published:** 2023-07-21

**Authors:** Mostafa Ghasempour, Akram Ghahramanian, Vahid Zamanzadeh, Leila Valizadeh, Laura A. Killam, Mohammad Asghari-Jafarabadi, Majid Purabdollah

**Affiliations:** 1 Students’ Research Committee, Tabriz University of Medical Sciences, Tabriz, Iran; 2 Department of Medical-Surgical Nursing, School of Nursing and Midwifery, Tabriz University of Medical Sciences, Tabriz, Iran; 3 Medical Education Research Center, Health Management and Safety Promotion Research Institute, Tabriz University of Medical Sciences, Tabriz, Iran; 4 Department of Medical-Surgical Nursing, School of Nursing and Midwifery, Shahid Beheshti University of Medical Sciences, Tehran, Iran; 5 Department of Pediatric Nursing, School of Nursing and Midwifery, Shahid Beheshti University of Medical Sciences, Tehran, Iran; 6 School of Health Sciences, Nursing, and Emergency Services, Cambrian College, Sudbury, ON Canada; 7 School of Nursing, Nipissing University, North Bay, ON Canada; 8 Cabrini Research, Cabrini Health, Malvern, VIC 3144 Australia; 9 Biostatistics Unit, School of Public Health and Preventative Medicine, Faculty of Medicine, Nursing and Health Sciences, Monash University, Melbourne, VIC 3004 Australia; 10 Department of Psychiatry, School of Clinical Sciences, Faculty of Medicine, Nursing and Health Sciences, Monash University, Clayton, VIC 3168 Australia; 11 Road Traffic Injury Research Center, Tabriz University of Medical Sciences, Tabriz, Iran

**Keywords:** Self-Presentation, Unsafe practice, Nursing student, Patient Safety, Qualitative Research

## Abstract

**Background:**

Maintaining patient safety is a practical standard that is a priority in nursing education. One of the main roles of clinical instructors is to evaluate students and identify if students exhibit unsafe clinical practice early to support their remediation. This study was conducted to identify self-presentation components among nursing students with unsafe clinical practice.

**Methods:**

This qualitative study was conducted with 18 faculty members, nursing students, and supervisors of medical centers. Data collection was done through purposive sampling and semi-structured interviews. Data analysis was done using conventional qualitative content analysis using MAXQDA10 software.

**Results:**

One main category labelled self-presentation emerged from the data along with three subcategories of defensive/protective behaviors, assertive behaviors, and aggressive behaviors.

**Conclusion:**

In various clinical situations, students use defensive, assertive, and aggressive tactics to maintain their professional identity and present a positive image of themselves when they make a mistake or predict that they will be evaluated on their performance. Therefore, it seems that the first vital step to preventing unsafe behaviors and reporting medical errors is to create appropriate structures for identification, learning, guidance, and evaluation based on progress and fostering a growth mindset among students and clinical educators.

## Background

Maintaining and promoting patient safety is a common responsibility among all participants in the healthcare system; Nurses have a significant impact on patient safety due to having the largest portion of all healthcare system employees [1]. Clinical education where students are placed in a practice setting is the cornerstone of undergraduate nursing programs and students may make medical errors while working with patients [2]. Educators need to balance the patient's right to receive safe care and create a suitable and safe environment for nursing students to learn [3]. Therefore, education that simultaneously maintains patient safety is a practical standard and main priority of educational institutions [3].

One of the main roles of clinical educators is ongoing evaluation of students and early identification of students with unsafe clinical practice [4]. For clinical educators, evaluating and identifying students with unsafe practices and how to manage them has always been challenging [5, 6]; Several factors can impair the ability of clinical educators to evaluate and identify students with unsafe practice. Some of these factors are the lack of a clear definition of the concept of unsafe clinical practice [7, 8], contradictory interpretations of unsafe student behaviors [9, 10], social pressures [11, 12], lack of clear frameworks [8, 12], fear of reprisal [13, 14], uncertainty in their decision to fail students [8, 15, 16], the added time needed to remediate students [17–19], and lack of support [11].

Before starting clinical placements, most educators do not have baseline knowledge about students and their performance. The behaviors and information that students display or provide about themselves during interactions with the instructor will therefore affect their evaluation and may cause instructors to make mistakes [20]. As a result, student's self-presentation may be used to conceal unsafe clinical practice from instructors; students who use this tactic want to give the educator the confidence to trust them and feel comfortable while doing the work [21].

Theorists describe self-presentation as a purposeful process to control the presentation of information about oneself in an effort to influence others; self-presentation is done to create, maintain, or modify an image of a person in the minds of "others" [22]. Self- presentation theory originates from Goffman's writings. In his opinion, people often interact with others similarly to a "presence on the theater stage". By saying the appropriate thing an actor (i.e. student) can shape a situation as they would like, enabling them to save face, gain respect, and move the interaction in whatever way the actor wants [23].

Two other theories, social influence theory, and interdependence theory, from the field of social psychology, discuss the importance of self-presentation tactics. Social influence processes are those tactics used by individuals to maximize desired rewards and minimize potential negative consequences associated with a given interpersonal interaction [24]. Also, according to the interdependence theory, social context can have strong effects on people's behavior. This theory highlights several structural dimensions that define a person's likely use of self-presentation tactics [25]. First, the future of one person depends on another person and is controlled by him. Research shows that to reduce the vulnerability associated with such contexts, people often try to create positive images to manage their self-image [25]. Second, the interests of the student who wants to receive the best evaluation possible conflict with the interests of the evaluator who wants to collect the most accurate information for evaluation. Interpersonal arenas with conflicting interests are theorized to always be associated with an increased use of self-presentation tactics [26, 27].

Dondolo and Chinyamurindi [28], found that people adjust how they portray themselves, their behaviors, and their attitudes during job interviews to convince panel members that they are well-suited for the relevant position and believe in the organization's values. Also, Patel et al. [29], in their study conducted among surgical residents, concluded that to convince their professors and peers that they have sufficient competence, self-confidence, and assertiveness to meet their expectations, they used strategies such as making up stories, silence, and avoiding asking for help. They mentioned that the main motivation of the participants for self-presentation is preventive action to create a positive background instead of a negative one. Also, they reported using impression management used effectively to achieve a more positive evaluation, more responsibilities for care, and greater freedom in learning and practicing clinical skills [29].

In Iran a Bachelor of Science in Nursing (BSN) program is four years (8 semesters) and according to its educational curriculum, more than 50% of student's time is spent in clinical placements. Clinical training is therefore important in shaping and creating clinical competence for nursing students as future nurses. The authors of this article include faculty members, most of whom are mainly involved in the clinical education of undergraduate nursing students. As clinical instructors, they have witnessed patient's safety being compromised many times during the training of students in clinical settings, making maintaining patient safety one of their main concerns. They were motivated to conduct a study analyzing the characteristics of students with unsafe clinical practices through the experiences of students, clinical instructors, and people involved in nursing education to support early identification of students with unsafe practice, timely remediation of their unsafe behaviors, and ultimately to maintain patient safety. The issue of students with unsafe clinical behaviors has been illustrated in previous studies. To our knowledge the concept of self-presentation and its possible role in unsafe clinical practice of nursing students has not been studied. As mentioned earlier, the concept of self-presentation as impression management has been studied among surgical residents in relation to job interviews [28], and in social media [29, 30]; in these studies, the safety consequences of self-presentation are only briefly mentioned. It also seems that considering the possible explanatory value of self-presentation across many interpersonal interaction research areas, understanding the tendency of students who have unsafe behaviors to use different types of self-presentation behaviors may be a useful tool to further understand nursing students with unsafe clinical practice. Therefore, we decided to investigate the components of self-presentation in nursing students to clarify the concept of nursing students with unsafe practices.

## Methods

### Study design

This study aimed to discover components of self-presentation in the clinical practice of students who are unsafe from the perspective of faculty members, preceptors, and nursing students using a qualitative research method. Streubert and Carpenter (2011) believe that qualitative research as a systematic and subjective approach leads to increased insight, understanding and awareness of human experiences. Therefore, to discover and explain dimensions of the phenomenon in question and reveal a deep understanding of the social world of the participants, an inductive qualitative research approach is most suitable [31]. Qualitative content analysis reveals the behavior, views, feelings and experiences of people and what is in the context of their lives. Among the three qualitative content analysis approaches, in the conventional method, categories are not determined in advance, but are extracted directly and inductively from the data text [32]. This approach to content analysis identifies the themes, and obvious and hidden patterns in the data through systematic classification of collected information and uses them to develop knowledge and gain new insights [33].

### Participant recruitment

Participants included faculty members, final year undergraduate nursing students, preceptors, and educational and safety supervisors of medical centers, which were selected through purposive sampling. The sample consisted of 19 persons, including 7 faculty members, 6 nursing students, 3 preceptors, 1 head nurse and 2 educational and safety supervisors. For inclusion in this study, we sought educators and managers with the most experience in teaching and working with nursing students. Also, Students needed to be enrolled in their 7th semester or higher to ensure they had sufficient exposure to clinical placements. Participants also needed to be willing to share their experiences. First, participants were simultaneously selected from clinical instructors and students; as the study progressed purposive sampling was used to obtain different points of view through identifying other participants with a goal of achieving maximum diversity (in terms of age, gender, level of education, type of hospital, and background and work experience in different departments). Continuous comparison and analysis of data and memos written at the same time as data collection guided the researcher toward the purposeful selection of the next participants. Sampling continued until no new information, categories and themes were obtained. The decision to achieve data saturation was made by re-examining the codes and their categories by members of the research team and two experts outside the research team. Two additional interviews were conducted to confirm data saturation.

### Data collection

The data for this study was collected through in-depth and face-to-face semi-structured interviews. The first three interviews were conducted by the first author, who asked the following research questions: "What is your own experience with safe/unsafe practices, and how do you feel about your own unsafe work? According to your experience, what characteristics can a safe or unsafe student have?".

The educators who participated in the study were asked to identify behaviors that they considered to be examples of unsafe student behavior in clinical environments and during internships. They were also asked to share their personal experiences as evaluators responsible for maintaining patient safety during internships.

Nursing students were also interviewed and asked to share their experiences with unsafe practices, as well as their observations of other people's behaviors. During the interviews, probing questions such as "What do you mean by excuse? Can you give an example? What was your own experience?" were used to analyze, guide, and confirm the statements made by the participants. They were encouraged to clarify and expand on the concepts they mentioned during the interviews.

The interview guide was refined based on four pilot interviews. Data analysis for pilot interviews was done in the form of continuous data comparison in parallel with the interview. After some themes emerged, they were used to refine the interview guide to collect more comprehensive and complete information. The pilot interviews were excluded from the study after analyzing and refining the interview guide. The time and place of all interviews were determined by participants. Interviews lasted between 40 and 60 min (Mean = 50 min). Data collection lasted from December 2021 to September 2022.

### Ethical considerations

This study was conducted as a part of a doctoral dissertation in Nursing after receiving the ethical approval code from Tabriz University of Medical Sciences (Ethical Code: IR.TBZMED.REC.1400.608). Before starting interviews, the researcher explained sufficient information about the research and the interview process, including audio recording, the confidentiality of information, and transcription of the participant's statements, and obtained written and informed consent to participate in the study. The interview started with some warm-up questions and then with a general question.

### Data analyses

After each session, all verbal and non-verbal messages, including silence and emphasis, were transcribed verbatim. Then, the text was read several times by the first and second authors (MG and AG) so that their immersion was done to communicate with the data. The written interviews were coded into semantic units at the word, sentence, and paragraph levels. The first author coded the interviews under the supervision of the research team. After coding, the coder preliminarily developed the study's main categories by understanding the similarities and differences in the extracted codes and continuously comparing them. Next, the coding's stability was checked using the Holsti method to ensure agreement between the coders (researcher and a coder outside the research team) [34]. A Holsti coefficient index above 0.7 was considered acceptable [35]. After ensuring sufficient consistency in coding, coding rules were applied to all parts of the text, and coding continued until no new codes, categories, and themes emerged. Research group meetings were held regularly during the coding process to check the content of the codes, ensure coding stability, and compile the categories. Finally, codes, categories, and subcategories were compiled using comparison, evaluation, feedback, and continuous interpretation. MAXQDA software, version 2010 (VERBI GmbH Company, Berlin, Germany), was used for data management during the analysis process. Table 1 provides an example of how the analysis moved from the data to the main concepts. Authors MA, AG, VZ and LV actively participated in the analysis and preparation of this part.

**Table 1:** The sample of data analysis for emerging of sub-category of defensive/protective behaviors

Category	Sub-category	Main concepts	Sample codes	Sample quote
Self-presentation	Defensive/protective behaviors	Excuses and justifications	Comparing your performance with other nurses to explain the cause of the error Justifying your wrong performance in the simultaneous injection of drugs	The student put all the medications in the microset, assuming that I didn't understand what he was doing. When I questioned him about why he arranged the medications in that way, he claimed that all nurses work like that and there is no issue with it
		Non-acceptance of error/Denial of error	Not accepting responsibility for mistake in the patient’s venipuncture Failure to accept the student's ignorance of the basic principles of venipuncture	I demonstrated to the student the proper places where the vein can be accessed for the patient, and I advised him to adjust the angle of insertion. However, he forcefully inserted the needle into the vein, and when I pointed out that the angle was not correct, he insisted that there was nothing wrong with his technique. He refused to acknowledge his mistake and showed a lack of knowledge in the proper technique for inserting a needle
		Underestimate of error	Underestimating one's own mistakes compared to the mistakes of others Underestimating the importance of error on the patient's prognosis by the student	Some educators in clinical settings focus excessively on minor details of our work, such as the process of preparing a syringe or cleaning the injection area, while neglecting to address more important issues, such as the non-sterile dressing of open wounds by ward nurses. Does it have a greater impact on patient outcomes or our work? I believe that our minor mistakes have little impact and can be overlooked
		Self-handicapping before performing procedures	Saying negative sentences before the procedure Excessive expression of anxiety by the student before the procedure	Before beginning to draw blood and even before applying the tourniquet, the student expressed a lot of anxiety. He claimed that the patient's vein was in a difficult location and that he was concerned about breaking it
		Projection	Blaming the lab staff for leaving bed side rail down Attributing one's own negligence to others	Our classmate lowered the side rail of the patient's bed, causing the elderly patient to almost fall due to loss of balance. Fortunately, the patient did not fall. When confronted about this, our classmate claimed that it was not his responsibility and that the laboratory staff had left the side rail down when they took the patient's blood. However, I was present in the same room at the time and did not observe anyone else enter the room to take the patient's blood

### Trustworthiness of data

In this study, the criteria proposed by Lincoln and Guba [36], including credibility, dependability, transferability, and confirmability were used to assure the trustworthiness of data. Through the long-term relationship with the participants, member checking, ensuring maximum diversity of participants, external checking, peer checking, and bracketing, the researcher became confident about the credibility of the data. In addition, audibility was supported by two experts who checked the analysis process (MA and LK).

## Results

The results of this study are a part of a Ph.D. in nursing thesis, which was conducted to identify the characteristics of students with unsafe clinical practices [37]. Table 2 shows some of the demographic characteristics of the participants in the study. In the main study, 3 categories of "insufficient readiness of students for safe clinical practice", "personal, social and professional factors" and "self-presentation" were extracted. Considering the extent of the results of the main study, this article only reports on the results obtained in the self-presentation category. This category has 3 main subcategories including defensive/protective behaviors, assertive behaviors, and aggressive behaviors (Table 3).

**Table 2:** Socio-demographic characteristics of participants

Participant number	Age	Sex	Level of Education/ Semester	Job Status	Clinical Experience (years)	Educational Experience (years)
1	40	Female	Ph.D. ^a^	Faculty Member	15	8
2	42	Male	Ph.D	Faculty Member	17	10
3	35	Male	M.Sc. ^b^	Faculty Member	8	6
4	49	Female	Ph.D	Faculty Member	13	15
5	53	Female	Ph.D	Faculty Member	20	15
6	38	Male	Ph.D	Faculty Member	11	8
7	54	Female	M.Sc	Faculty Member	20	18
8	24	Female	Nursing student / 8^th^	Student	-	-
9	30	Male	Nursing student / 7^th^	Student	-	-
10	24	Female	Nursing student / 7^th^	Student	-	-
11	23	Male	Nursing student / 6^th^	Student	-	-
12	34	Male	Nursing student / 8^th^	Student	9 (LPN)^c^	-
13	23	Female	Nursing student / 7^th^	Student	-	-
14	30	Male	M.Sc	Preceptor/nurse	7	2
15	37	Female	M.Sc	Preceptor/nurse	12	3
16	35	Male	M.Sc	Preceptor/nurse	10	3
17	46	Female	BSN ^d^	Head nurse	21	-
18	47	Male	M.Sc	Educational supervisor	22	10
19	35	Female	BSN	Safety supervisor	10	-

^**a**^Doctor of Philosophy

^**b**^Master of Science in Nursing

^**c**^Licensed practical nurse

^**d**^Bachelor of Science in Nursing

**Table 3:** Illustration of the categories, subcategories, and attributes of the topic

Category	Subcategories	Attributes
Self-presentation	1. Defensive/protective behaviors	1–1. Excuses and justifications
		1–2.Non-acceptance of error/Denial of error
		1–3.Underestimate Of Error
		1–4.Self-handicapping before performing procedures
		1–5.Projection
	2. Assertive behaviors	2–1.Ingratiation/flattery
		2–2.Directing the educator's mindset by magnifying their abilities
		2–3.Entitlement to receive rewards
		2–4.Modelling your behavior for peers
	3. Aggressive behaviors	3–1.Exaggeration in negative evaluations of others' abilities / Humiliation of peers
		3–2.Cast doubt about the competence or credibility of the educator

### Defensive/protective behaviors

According to participants, defensive self-presentation is used when an error or negligence occurs, and the student interprets it as a factor in jeopardizing their professional identity. Therefore, a person tries to correct their damaged identity or reduce the negative effects of the event through various methods.

This subcategory includes the following 6 attributes:

#### Excuses and justifications

According to participants, making excuses and justifying one's actions may be one of the defensive and protective self-presentation tactics. The participants reported that the students used various excuses and justifications such as lack of access to patients' medical records, lack of knowledge of the place and time of the internship, lack of knowledge of the institution's rules regarding standards, referring to the performance of others in the same situation to defend their unsafe behavior. Both students and educators believed that irrational excuses of students who do not accept responsibility for unwanted events can be one of the components of defensive/protective behaviors. One of the preceptor participants says:

*“I tell the student that it is written here to give the medicine based on the potassium level, why did you give him the medicine when the potassium level is high, he says that the patient's file was with the intern and he did not give it to me.”* (P: 4)

In many situations of error, providing overriding and important reasons to justify the error, along with accepting responsibility for it, can be one of the defensive behaviors of students to preserve their image and professional identity. As one of the students says:

“*Once, our groupmate mistakenly connected washing serum instead of normal saline serum, although he finally accepted his mistake, he kept saying that I am sure that he was there and that he had left it from the previous shift to connect it to the patient, I was busy and I forgot to take it.”* (P: 9)

#### Non-acceptance of error/denial of error

According to participants, not accepting responsibility for committing a mistake and/or denying it by the student is one of the tactics used by students to defend their professional identity and reduce potential negative consequences of the mistake. In the early stages of revealing the error, they try to deny or accept the error by using different methods such as using lies, hiding the error, and not taking responsibility for having a role in the occurrence of the error. One of the preceptors says:

“*According to the order, 3 cc of the 10cc vial of magnesium sulfate should have been injected for the patient. Once the patient flushed and had palpitations, the patient said that this lady (pointing to the student) poured the entire ampoule into my micro set; while the other nurses were injecting a small amount of it. And I felt bad after that. I said, did you give him too much medicine? But the student didn't accept it at all*.*”* (P: 14)

#### Underestimate of error

Explaining the low importance of the error may be another self-presentation tactic. Participants reported that to reduce the consequences of their medical errors, students try to underestimate the errors that occurred during drug administration, maintenance of a sterile field, and performance; students use methods such as valuing errors from least important to most important and expressing memories of similar cases and the absence of problems for previous patients. For example, one educators says:

“*I tell the student, why didn't you remove the air inside the serum set before injection, he says that these air bubbles do not exceed a few cc in 24 hours, it is not very dangerous, and the body will solve this amount by itself.”* (P: 2)

From a student perspective:

“*Some educators pay a lot of attention to opening the syringe and disinfecting the injection area, while we saw that the personnel did non-sterile dressing of the wound, which could have a greater impact on the patient's prognosis?! I say our mistake has much less impact and can be ignored.”* (P: 13)

#### Self-handicapping before performing procedures

Presenting oneself weakly before nursing procedures is one of the defense tactics used by students to maintain their identity and manage the perceptions of their instructors. Students try to adjust the expectations of others towards themselves; and justify their possible failure in performing procedures by making negative statements, exaggerating the obstacles and difficulties of the procedure, expressing their excessive concern and citing problems related to the patient's condition (such as difficult-to-locate blood vessels or patient complaints). One of the participants said:

“*Before inserting the Intravenous Line, the student says, I can't see the vein, and after inserting the catheter, I lose the vein. Students do this to make the situation look difficult. They prepare the educator for their possible failure and moderate the educator's expectations.”* (P: 16)

#### Projection

Projection may also be one of the characteristics of defensive self-presentation among students exhibiting unsafe clinical practice. Some participants expressed experiences in which students tried to blame others (for example, unit personnel, patients, other peers, etc.), and introduce them as the main cause of errors; So that they can protect themselves from creating a negative image in front of others or reduce the negative consequences of their mistakes. One of the students says about making a medication error:

*“The nurse gave me the syringe filled with phenytoin; I thought that I have to infuse the whole drug for the patient. I poured all the medicine into the microsite, then she said what did you do with the rest, I said, I injected it all. The doctor's order was 100 mg and I had been given 150 mg. It was not my fault; the nurse was at fault.”* (P: 11)

### Assertive behaviors

According to participants, assertive behaviors refer to preventive behaviors that students who are unsafe perform to create specific identities and obtain secondary benefits in front of educators. In general, the interpersonal goal of assertive behavior is to develop or create a specific identity.

This subcategory includes the following 4 attributes:

#### Ingratiation/flattery

During interviews, participants shared that students with unsafe performance, hide their weaknesses and mistakes to gain favor by attracting the interest of the instructor. This ingratiation may manifest itself in various forms such as increased communication, flattery, excessive conformity with the educator's opinions, and doing favors or giving gifts. According to participants, this behavior can influence the instructor and lead to a sense of trust in the student. However, excessive trust in the student can negatively impact the monitoring and evaluation of their actions and potentially result in safety incidents for both the patient and the student. One student explains:

“*Our classmate used to flatter himself in every field and always confirmed the educator's opinions. He wanted the coach to ignore his absences and late arrivals; so that his grade does not decrease.”* (P: 8)

Another student says that:

*“We had a groupmate, wherever we went for the internship, to get more marks, he praised that educator more than enough, and he said that he learned many things there; and he introduced the educator as his best educator. He was exaggerating a lot.”* (P: 12)

#### Directing the educator's mindset by magnifying his abilities

Magnifying and exaggerating one’s own abilities may be one of the characteristics of self-presentation among students with unsafe clinical practice. Some students direct their instructor’s mindset to cover their weaknesses and gain confidence. Diverting the instructor’s attention creates a safe margin for themselves and aims to create a more positive student image by exaggerating their abilities (especially practical abilities) and downplaying their weaknesses. One educators says:

*“Some students like to focus and emphasize more on the parts they know more about; to show themselves to their educators and friends. On the contrary, they cover their weaknesses so that they are less visible.”* (P: 3)

#### Entitlement to receive rewards

Expressing undue entitlement to receive a reward from a student with unsafe clinical performance with the aim of impressing the instructor is one of the other characteristics of assertive self-presentation. According to participants, some students try to show their work more prominently than their peers in order to impress their instructors and attract attention, receive encouragement, and earn higher grades. In these situations, the student makes unrealistic claims about having more responsibility and contributions to successes, regularly referring to the things they did during a clinical placement but the educator did not notice. Participants felt that these actions can unrealistically portray the person as a student with scientific and clinical abilities in the eyes of the instructor and lead to instructor negligence when supervising that student’s performance. One student explained:

*“We had an internship where we had to prepare a pamphlet for patients. Our friend showed the part that he had worked on to the educator and emphasized that this part was his work, and he used some references. Mostly, his tactic was to make his work more important and prominent to get a better grade.”* (P: 10)

#### Modelling your behavior for your peers

It seems that according to the experience of the participants, modelling their behavior for peers by students with unsafe clinical practice can be one of the characteristics of assertive behavior for self-presentation. The nature and purpose of most self-presentation behaviors are based on impressing and gaining the instructor's trust. Most of the students who have experience in clinical work have already experienced the skills taught by the instructor to the students. Therefore, they have too much self-confidence about their performance, as a result, they are less careful than the beginner students and they omit some basic steps of nursing procedures. Their approach to work was more experimental and relied on non-scientific techniques. These students try to influence the instructor to ignore their absences and tardiness, failure to prepare the assignments requested by the instructor, participate in more invasive procedures in clinical environments and perform them informally and teach other students incorrectly. In this case, one of the participating instructors said:

*“Students who have work experience, especially if the instructor is less experienced, in these cases the student dominates and teaches all the wrong techniques to other students. For example, I tell the student to infuse Gentamicin into the Microset. I ask the student where is the patient’ Microset? He says that Mr. X said, to dilute the medicine with 10 cc and inject it directly.”* (P:15)

### Aggressive behaviors

According to participants students use the method of dominating or belittling others to make themselves look good, which was labelled aggressive tactics. By attacking others and portraying themselves as competent, they try to perpetuate their desired self-image. This subcategory includes the following 2 attributes:

#### Exaggeration in negative evaluations of others' abilities / humiliation of peers

Participants believe that exaggerating negative and critical evaluations of others' abilities can be one of the aggressive behaviors of students with unsafe clinical practices to reduce the consequences of medical errors for themselves. Since educators usually use comparison for judgment and evaluation, one method used by students who exhibit unsafe practice is to convey themselves more positively than others by diminishing the work of peers. They enact their intentions through making exaggerated negative statements about the abilities and mistakes of their peers. One of the participants mentions:

*“One person's name came up, I praised his work, one of the students started talking behind his back, destroying him and saying something that he had given the wrong medicine to the patient, and if the educator didn't notice, the patient might have died.” (P: 6)*

#### Cast doubt about the competence or credibility of the educator

Based on the participants' experiences, instead of accepting criticism, students who practice in unsafe ways try to reduce the intensity of criticism by being aggressive and doubting the competence of the criticizing source (i.e., questioning the educator's competence). This tactic can include accusations of the evaluator's incompetence, inexperience, and bias. One of the participants says:

*“The student had a medication error, I tell him that it could have led to bad side effects for the patient, instead of accepting the criticism, he tells me in a bad and aggressive tone that you don't have enough experience to teach us.” (P: 1)*

## Discussion

### Main findings

This research is the first study that was conducted to identify components of self-presentation in students with unsafe clinical practice. The results of the study showed that "defensive/protective behaviors", "assertive behaviors" and "aggressive behaviors" of students are key elements of self-presentation. In general, nursing students who practice unsafely may resort to various forms of self-expression to impress clinical instructors, gain their trust, and achieve better grades, even if they have deficiencies in their abilities and make medical errors. However, these tactics can lead to the development and maintenance of an unrealistic identity, which can have serious negative consequences. Such tactics can disrupt the safety of the student's performance, increasing the risk of harm to themselves, staff, patients, and peers. Therefore, educators must remain particularly vigilant to identify these self-preservation tactics in an effort to support student success and safety in clinical settings.

### Comparison with other studies

In general, these findings are consistent with the results of studies conducted with medical students [29, 38], or in other fields [28, 39–41]; These findings support that in situations where the student is being evaluated, it is possible that students may selectively show behaviors with the intent of managing the perception of others. Another study also found that learners typically react to formal evaluation by making changes in their clinical practice and speech to please the observer; they change their practice from "patient-centered care" to "practice-centered care" [42]. It is not always possible to attribute a specific style of self-presentation to a person, because people with different personality traits use different styles at different times and in different situations [43]. However, the findings of the present study showed that defensive/protective behaviors may be an important component for identifying students with unsafe clinical practices. Lee et al. [44], have also identified defensive/protective self-expression as part of self-presentation behaviors when people assume that others are aware of information that portrays them negatively (i.e., making mistakes), self-presentation efforts may increase. For example, after receiving unfavorable feedback from the evaluator, people may make compensatory self-presentations to repair their damaged image [22, 45].

Nursing students may believe that consequences of making medical errors will be harmful to their program progression. Therefore, fear of error reporting and concealment of errors have been reported among nursing students [46]. On the other hand, many social conditions such as social anxiety and fear during interaction with others can be related to defensive self-presentation [44, 45, 47]. The results of the systematic review show that nurses refuse to report their medication errors due to fear of being blamed, loss of reputation and dignity, loss of position, and fear of being considered incompetent by colleagues [48]. Also, in another study, self-preservation and fear of adverse consequences of mistakes such as disciplinary actions, reprimands, and legal actions were reported as reasons for reluctance to disclose mistakes [49]. Participants in the current study also reported the fear of losing their image in the eyes of their teachers and friends, fear of negative evaluation, and the educational and legal consequences of errors, which led them to use various methods such as concealment or irrational excuses, rationalization, denying the error, underestimate of errors, self-handicapping, and projection to defend their professional identity or reduce the negative effects of mistakes.

Findings from the current study showed that assertive and aggressive behaviors are perceived as defining components of self-presentation. A review of the literature shows that many people believe that they are better than others in many ways and behave accordingly [50]. People may convey self-superiority beliefs to others by claiming that they are "better than others". In some literature, these assertive behaviors are listed as "explicit self-superiority claims" and "implicit self-superiority claims" [51]. In the present study, students who acted in unsafe ways tried to portray themselves as better than others by behaviors such as ingratiation, exaggeration in their abilities and knowledge, self-promotion, considering themselves entitled to rewards, and acting as role models. In most cases participants considered these behaviors to be fake and inconsistent with reality. The findings of another study also showed that observers evaluated claimants explicit statements of superiority more unfavorably than others [51]. In contrast, Rafiee et al. [20], found that students perceived that educators give higher grades to students who present themselves falsely during clinical evaluations. Also, in the field of organizational interviews researchers have shown that to make themselves worthy, applicants use self-promotion, ingratiation, exemplification, and sometimes threatening behaviors [41, 42]. In the present study, not all self-presentation behaviors are designed to provide a desirable identity. Students may have aggressive or projective behaviors to achieve interpersonal goals such as better evaluation and showing themselves innocent in the event of a mistake. In line with the present study, the findings of other studies show that people may use negative evaluation of others [44], criticism of the other person [43], aggression [43, 44], and even creating fear and intimidation [44] to allow certain negative impressions to be formed about them in order to disguise other important negative impressions or achieve other more important goals or long-term benefits [52].

Students are significantly concerned that others will not perceive and evaluate them in favorable ways. Therefore, proactive and assertive self-expression appears more in situations of judgment and evaluation. Since most people consider the judgments and evaluations of others to be negative and anxiety-provoking, they may intensify the preventive transfer of favorable information about themselves before receiving any humiliating feedback [22]. We expect people to proactively assert power in evaluative situations before disappointing performance in a future evaluative situation [38]. In line with the findings of the present study, surgical residents in the study of Huffman et al. [53], identified their "audiences" and changed their thinking and practice based on it. They tended to portray their competencies and avoid weaknesses and uncertainty [38]. The results of a scoping review also showed that students in clinical situations avoid showing their deficiencies or deny them as an act of self-preservation [54]. Participants in Patel et al.’s study also experienced less willingness to ask questions due to the fear of spoiling their image; they wanted to maintain their pride, which was an obstacle to their learning. Also, anxieties related to judgment and evaluation have a negative impact on student mental health and decision-making ability, which has disrupted the provision of quality and safe care for patients [38, 53].

Within the present study, students sometimes learn some implicit expectations of themselves that are not explicitly stated in the curriculum through the hidden curriculum and shape their behavior based on them. Some participants have stated that asking for help and asking questions may be included in the curriculum, but some informal aspects of the curriculum present their questions as a student's weakness. Therefore, they try to preserve their identity by refusing to ask questions or by showing defensive or assertive behaviors about their practice. According to previous studies, explicit and implicit/hidden curricula may be inconsistent with each other. In this situation, the hidden curriculum is likely to have a stronger impact on the development of professional values and behaviors [55].

Fear of reporting mistakes to an educator is a major problem in clinical education because open and honest learner-educator relationships are needed to preserve patient safety. Dishonesty is consistently ranked as one of the most unsafe actions of a student [7, 16, 56–59]. To promote honesty when mistakes are made, educators need to take intentional actions towards building safe spaces to disclose errors. This safe space may mean acknowledging that unsafe student behaviors may be a result of perceived barriers within the clinical environment or program [60].

The current study relied on open questions and in-depth interviews of participants, which allowed the effective components of self-presentation among students who act in unsafe ways to be identified in an exploratory way. However, demographic factors that may influence self-presentation such as gender, race, ethnicity, and geographic region were not investigated; the social and cultural conditions of a society may affect the interpersonal interactions of people. Therefore, the potential role of these factors should be investigated in future studies. In addition, students may not have been completely honest in their interviews about unsafe actions that they may have been involved in due to social desirability. Researchers strived to mitigate this risk by ensuring participants of the confidentiality of the interviews. This study, like other qualitative studies, should not be generalized. Instead, readers may assess the potential transferability of these findings to their own context.

Despite these limitations, identifying the components of self-presentation in nursing students paves the way for making tools for measuring self-presentation behaviors. The results of this study can help create and expand future studies in the field of identifying nursing students with unsafe practices.

## Conclusions

When students anticipate that they will be evaluated on their future practice, they may choose to take action to shape their presentation proactively and assertively or simply wait until an issue has occurred and, if necessary (e.g., if failed to do the job), engage in defensive and protective compensatory self-presentations. Therefore, it seems that the first vital step to prevent inappropriate behavior of students due to unsafe practices is to create appropriate structures for supporting all students to be honest with an educator. Proactive actions to prevent unsafe student behaviors include creating systems for identification, remediation, guidance, and supportive student evaluation based on their progress. Clinical educators require support when interpreting and responding to nuanced student behaviors that may pose safety risks [6], particularly when students are disguising their weaknesses. Discussion with students about how important honesty is for patient safety and their learning may be used to foster a growth mindset among students and educators. Using appropriate relationships for mentoring students and competency frameworks to guide and develop learner abilities may further help to prevent inappropriate self-presentation behaviours. By creating and fostering a suitable cultural atmosphere, students should be encouraged to accept their mistakes, recognize the shortcomings of their knowledge and practice, and use the opportunities to learn more, instead of resorting to the discussed tactics. For future research, it is recommended to conduct more quantitative and qualitative studies across various societies and cultures, focusing on students from different medical fields, to identify the concept of self-presentation and its components. These studies can explore why and how students use self-presentation tactics, as well as the impact of these tactics on interpersonal relationships, patient safety, and unsafe practices. Furthermore, the findings of this study can aid other researchers in developing tools to identify self-presentation behaviors among students who practice unsafely.

## Data Availability

The data that support the findings of this study are available from the corresponding author upon reasonable request.

## Abbreviations

BSN: Bachelor of Science in Nursing
LPN: Licensed practical nurse
M.Sc.: Master of Science in Nursing
Ph.D.: Doctor of Philosophy
